# Autologous Platelet-Rich Plasma for Healing of an Oncologic Dehisced Wound

**Published:** 2013-11-04

**Authors:** Samin Alavi, Fatima Malek, Peyman Eshghi, Hossein Arzhangian

**Affiliations:** Pediatric Congenital Hematologic Disorders Research Center, Mofid Children's Hospital,Shahid Beheshti University of Medical Sciences, Tehran, Iran.

**Dear Sir,**

Autologous platelet-rich plasma (PRP) consists of cytokines, growth factors, chemokines, and a fibrin scaffold derived from the patient’s blood. The mechanism of action for PRP gel is considered to be the molecular and cellular induction of normal wound healing responses similar to that seen with platelet activation.[1,2] There are multiple indications of PRP therapy, but there are few controlled trials to verify its efficacy. Here, we report an adolescent with an oncologic wound which was dehisced following radiation therapy.

A 13-year-old boy, a known case of a soft tissue sarcoma of the extremity, was referred to the pediatric oncology clinic because of dehiscence of his surgical wound following radiation therapy. He had a massive tumor of upper extremity for which a wide local excision was done. Radiation therapy was performed within less than one month following surgery which lasted for about 5 weeks. The surgical wound was a curved scar in the antecubital area. Firstly, the dehiscence was tried to be managed by clean wet dressings for about 2 weeks. However, due to lack of response he was treated with PRP gel (Fig. 1A, 1B). The patient’s wound responded dramatically and healed in two weeks (Fig. 2A, 2B).

**Figure:**
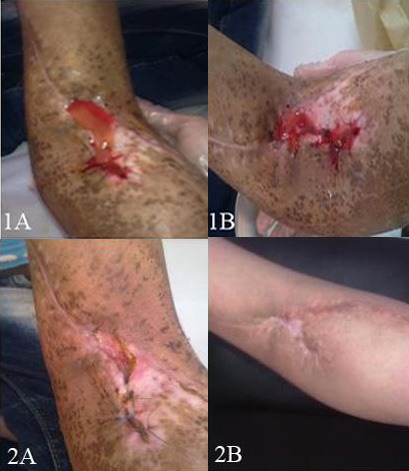
Figure 1A, 1B: Application of patient’s gel on the wound at place of dehiscence. 2A: wound healing 2 weeks later and 2B: 1 month later.

During the last two decades, there was a burst through PRP therapy in tissue repair. Growth factors released from activated platelets initiate and modulate wound healing in both soft and hard tissues. A variety of potentially therapeutic growth factors were detected and released from the platelets in significant levels in platelet-rich plasma preparations. Release of these growth factors into the site of wound injuries could expedite wound healing.[3] The very first application of PRP was in the field of musculoskeletal system.[1,4] McAleer et al have also reported their experience with concentrated autologous platelet-derived growth factors in healing of chronic lower-extremity wounds which was evaluated in 24 patients with 33 lower-extremity wounds.[5] Wound closure and complete epithelialization was achieved in 20 wounds.[6] Villela et al carried out a systematic review in 2008 and found five studies on ulcers of diabetic etiology. The results of meta-analysis showed that PRP had positive effects on the healing process (95% CI: 2.94-20.31).[6] We used PRP for a young boy with non-healing surgical wound as a result of radiation therapy and achieved success. PRP thus may be tried in such conditions.

## Footnotes

**Source of Support:** Nil

**Conflict of Interest:** None declared

